# A mindful approach to physician self-care

**DOI:** 10.4102/safp.v66i1.5836

**Published:** 2024-01-30

**Authors:** Janine M. Kirby, Peter D. Milligan, Hofmeyr H. Conradie, Belinda M. McIntosh

**Affiliations:** 1 Dr Janine Kirby, Private Practice, East London, South Africa; 2 Department of Psychiatry, College of Health Sciences, University of KwaZulu-Natal, Durban, South Africa; 3 Department of Family Medicine, Faculty of Medicine and Health Sciences, Stellenbosch University, Stellenbosch, South Africa; 4 Department of Psychiatry, Faculty of Health Sciences, Tintswalo Hospital, Acornhoek, South Africa

**Keywords:** self-care, burnout, systems failure, presence, perspective, self-compassion

## Abstract

There has been an increasing awareness of the importance of physician mental health. Several South African studies show a high prevalence of burnout among doctors. Burnout is characterised by three components: exhaustion, depersonalisation, and a sense of a lack of efficacy. Burnout is a result of both external and internal pressures. While lifestyle modification is essential, mindfulness-informed programmes promote self-regulation and resilience. Mindfulness programmes comprise three components: present moment awareness, perspective-taking and wisdom, and compassion. Physician wellness begins with individuals recognising the need of self-care and giving themselves permission to prioritise this. Ongoing identification of self-care needs and acting compassionately to address these needs is essential.

There has been an increasing awareness of the importance of physicians’ mental health.^1^ This was especially highlighted during the coronavirus disease 2019 (COVID-19) pandemic when levels of burnout dramatically increased. A study published by the Mayo Clinic in 2022, showed that 62.8% of physicians fulfilled at least one of the criteria (see below) for burnout, while the number was 38.2% in 2020.^2^ In South Africa, various support structures, such as the HealthCare Workers Care Network,^3^ were established to support medical staff. The crisis has passed but the need that was highlighted during the pandemic for ongoing care for medical staff, remains.

Several South African studies show a high prevalence of burnout among doctors ranging between 59% and 100%^4,5^; while in a systematic review of burnout among healthcare providers in sub-Saharan Africa, the prevalence of burnout among doctors ranged between 59% and 81%.^6^

Levels of burnout were higher among rural doctors,^4,5^ female doctors,^4,5,7,8,9^ and junior doctors.^5,8,9,10^

## Definition of burnout

Burnout is characterised by three elements as defined by Maslach et al.^11^

### Exhaustion specific to the work environment

Occupational exhaustion (burnout) is typically connected to a relationship with work that is perceived as difficult, tiring, and stressful. Maslach sees this as different from depression, as it is likely that the symptoms of burnout would be reduced outside of the work environment.^12^

### Depersonalisation or compassion fatigue

This is characterised by a loss of regard for others (patients and colleagues), keeping an emotional distance, which can be expressed through cynical, derogatory remarks, and even callousness.^12^

### Sense of a lack of efficacy

The personal accomplishment assessment is a feeling that acts as a ‘safety valve’ and contributes to bringing about a balance if occupational exhaustion and depersonalisation occur. It ensures fulfilment in the workplace and a positive view of professional achievements.^12^

The symptoms of burnout, if left unchecked, can cascade into anxiety, depression, post-traumatic stress, and even suicidal ideation.^1^ Burnout is associated with poor communication with patients, a reduction in the quality of the doctor–patient relationship, doctors struggling to meet work demands, and higher incidences of mistakes.^1^

## Why do doctors burn out?

### No time to rest

In an ideal work environment, times of being stretched by stressful, complex medical problems should alternate with periods of relative calm and rest.^1^ However, physicians work in a toxic milieu of both internal and external pressures.

### The patient comes first

The external pressures include working with large numbers of patients, the complexity of medical record-keeping, dealing with difficult psychosocial issues, long working hours (in the United States 25% of doctors report working 60 h – 80 h per week), and a lack of work–life integration.^13,14,15^

### Systems failure

One of the major causes of stress and burnout in health workers is systems failures. These include supply chain problems, non-availability of critical medicines, staffing problems, and increasing clinical loads. This coupled with inadequate managerial support can massively increase the pressure on health workers.^1,13,14^

### Do not show weakness

Medical education is long and arduous. Students are taught to perform, work hard, and take on responsibilities. Physicians are also trained to be able to critically analyse problems. This can enhance the inner critic and boost the predisposition to perfectionism.^13,14,16^

### Competition for attention

Tim Wu in his book *The Attention Merchants* writes ‘Over the coming century the most vital human resource in need of conservation and protection is likely to be our own consciousness and mental space’.^17^ There is an abundance of continuing professional development (CPD) on-line talks, in-person presentations and conferences, journals, and study groups that compete for doctors’ attention and time. Social media companies have intentionally designed their products to be addictive,^18^ meaning that empty moments are filled scrolling through Instagram, Facebook, X (formerly known as Twitter), YouTube, and TikTok. This added time drain makes it difficult to find and prioritise time for self-care and reflection.

## Self-care

Rachel Remen,^19^ a medical doctor and storyteller writes:

The expectation that we can be immersed in suffering and loss daily and not be touched by it is as unrealistic as expecting to be able to walk through water without getting wet.

Doctors are familiar with lifestyle modifications that help to mediate stress. These include scheduling down time, sleeping well, maintaining social connections, exercising, and eating well.^2^ These strategies are essential and helpful in supporting stressed healthcare workers; however, they do not address the emotional states and thoughts associated with compassion fatigue and loss of meaning.

The impact of mindfulness-based interventions (MBIs) on physician wellness has been widely studied and published.^20,21,22,23^ Contemplative researchers have shown that mindful body-awareness practices improve emotional regulation and resilience. It has also been noticed that people who complete a mindfulness-informed programme significantly improve their level of self-care.

## Mindfulness-informed programmes cover the following areas

### Present moment awareness

Mindfulness practices encourage participants to become aware of the present moment.^24^ This awareness is practiced with daily formal meditations (such as a scanning though the body, tuning in to the physical sensations that are present, or focusing on the breath) as well as informal shorter practices (e.g. intentionally taking a few slow breaths, attentively washing hands, or walking mindfully between patients [see Box 1]).

BOX 1 Short present moment awareness practices.

***Short practices***

**Breathing**

Take a deep and slow breath in to a count of 4, hold it to a count of 4, exhale slowly to a count of 4, repeat 3 times.

**Washing hands**

When washing hands, become aware of the temperature and feel of the water, the smell and sensation of soap, massaging and caring for the hands, which are so integral to the work of caring and healing.

**Walking**

Dropping the attention down to the feet on the ground, noticing the movement of each foot as it lifts and lands, the transfer of weight from side to side.

### Perspective-taking and wisdom

This is developed through becoming aware of and labelling what is happening.^24^ Each experience is accompanied by a bodily sensation, an emotional component, and attending thoughts. Participants are invited to elect to come off automatic pilot, objectively appraising the experience. This awareness is sharpened during small and large group discussions as participants become more aware of their unconscious patterns, beliefs, triggers, and reactions.

This intimate tuning in means that participants can choose *how* to be present, rather than *what* to do (see Box 2).^25^

Box 2 STOP, an acronym for tuning into the present.

**Figure:**
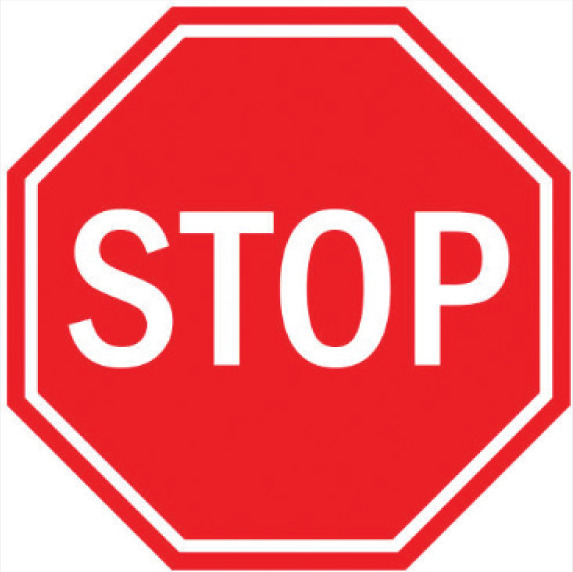


**STOP** ^25^

The acronym STOP is a practice that can be sprinkled through the day.

**S** Stop, take an intentional pause.

**T** Take a few breaths.

**O** Open up to and observe what is happening in your body, thoughts and emotions

**P** Proceed, choosing a path that is wise and compassionate.

*Source*: Rosenbaum E. The heart of mindfulness-based stress reduction; A MBSR guide for clinicians and clients. Eau Claire WI: PESI Publishing & Media; 2017

As an example, seeing a drunk patient arrive towards the end of a long casualty shift, results in an instant feeling tone in the body – which could range from empathy to dread to disgust to anger – with a resultant cascade of thoughts, emotions, and bodily sensations. Becoming aware of this gives the doctor the ability to choose how to be present; the situation cannot be changed, but the healthcare worker can choose a path of wisdom and compassion.

Holocaust survivor and psychiatrist Viktor Frankl wrote:

Everything can be taken from a man but one thing: the last of the human freedoms – to choose one’s attitude in any given set of circumstances, to choose one’s own way.^26^

### Compassion and self-compassion

Constructive practices such as compassion and self-compassion^24^ are cultivated, recognising that doctors are trained to be critical and constantly evaluate the performance of themselves and their colleagues.

Psychologist Kristin Neff^27^ educates on the power of practicing self-compassion, defining it as ‘giving ourselves the same kindness and care we would give a close friend’.

She notes that self-compassion has three elements^25^:

- *Self-kindness versus self-judgement*: People have a tendency to be kind and caring to others, but self-critical and harsh towards themselves.
- *Common humanity versus isolation*: When stressed, there is a notion that something is wrong with only me, rather than recognising that suffering and difficulty are universal.
- *Mindfulness versus over-identification*: Mindfulness practices invite an objective balanced appraisal, rather than ruminative over-thinking.

The following themes are common in participants who have undertaken a mindfulness-informed intervention^27,28^:

1. Sharing personal experiences from medical practice with colleagues reduced professional isolation. The sense of not feeling alone reinforced the need for community.
2. Mindfulness skills improved the participants’ ability to be attentive and listen deeply to patients’ concerns, respond to patients more effectively, and be more flexible.
3. Developing greater self-awareness was positive and transformative. Participants became aware of their own distress and adaptive and maladaptive strategies. This awareness can inform better choices in how to respond to the situation differently, wisely, and kindly.
4. Physicians were better able to self-regulate. Resilient doctors were able to ‘bounce back’ after adversity, owning up to limitations and mistakes and able to set boundaries.
5. Participants became more aware of the communities in which they practiced and developed a greater sense of accountability, service, and responsibility.

A recurring theme is that participants found it hard to prioritise time for self-care.^2^ It is difficult to get doctors to sign up for programmes, despite the benefits. Some programmes are self-directed and online to alleviate the problem of time constraints. However, this removes the benefit of participants connecting with each other and not feeling isolated.

While physician self-care interventions are important, the need for systems change is also crucial. Some interventions are focused at leaders in the healthcare sector,^2,21^ empowering them to make decisions that not only prioritise quality of care but also the well-being of healthcare professionals.

The need for physician self-care has become essential. The question regarding how to address this need has spawned many programmes and research. Mindfulness-informed interventions have been shown to improve levels of compassion, resilience, and emotional regulation. The research also points to important workplace outcomes such as doctors who listened more attentively, made fewer mistakes, and had an increased sense of service and meaning.
